# Live cell dynamics of the NF-Y transcription factor

**DOI:** 10.1038/s41598-021-90081-1

**Published:** 2021-05-26

**Authors:** David G. Priest, Andrea Bernardini, Jieqiong Lou, Roberto Mantovani, Elizabeth Hinde

**Affiliations:** 1 School of Physics, University of Melbourne, Melbourne, VIC Australia; 2 Department of Biochemistry and Molecular Biology, Bio21 Institute, University of Melbourne, Melbourne, VIC Australia; 3 Dipartimento di Bioscienze, Università degli Studi di Milano, Via Celoria 26, 20133 Milan, Italy

**Keywords:** Biochemistry, Biophysics, Cell biology, Molecular biology

## Abstract

Transcription factors (TFs) are core players in the control of gene expression, evolutionarily selected to recognise a subset of specific DNA sequences and nucleate the recruitment of the transcriptional machinery. How TFs assemble and move in the nucleus to locate and bind their DNA targets and cause a transcriptional response, remains mostly unclear. NF-Y is a highly conserved, heterotrimeric TF with important roles in both housekeeping and lineage-specific gene expression, functioning as a promoter organiser. Despite a large number of biochemical, structural and genomic studies of NF-Y, there is a lack of experiments in single living cells; therefore, basic assumptions of NF-Y biology remain unproven in vivo. Here we employ a series of dynamic fluorescence microscopy methods (FLIM-FRET, NB, RICS and FRAP) to study NF-Y dynamics and complex formation in live cells. Specifically, we provide quantitative measurement of NF-Y subunit association and diffusion kinetics in the nucleus that collectively suggest NF-Y to move and bind chromatin as a trimeric complex in vivo*.*

## Introduction

Orchestration of gene expression underlies the differentiation of cells and development of organisms, and the corruption of transcriptional regulation is a central feature of diseases such as cancer^1^. Transcription is ultimately governed by Transcription Factors (TFs) that bind to short (typically 4–8 bp), specific DNA elements in the promoters and enhancers of their target genes. These TF motifs are present in millions of copies throughout genomes, yet TFs are remarkably selective, only targeting a few thousand potential sites. Thus, how TFs efficiently locate and bind their correct DNA target sites in the nucleus has been a central question^2^. Packaging of genomic DNA into chromatin by nucleosomes blocks access to most DNA binding proteins to maintain a ‘silent’ chromatin state^3^. However, a class of TFs known as ‘pioneers’ can bind their DNA motifs in silent chromatin and initiate transactions on DNA by recruiting secondary factors such as chromatin remodelers, co-activators and histone-modifying proteins^4–6^. Binding of pioneer factors can also destabilise nearby nucleosome(s) and release DNA motifs for non-pioneer TFs, often directionally along chromatin^7^.

Measurement of TF dynamics in live cells provides important insights into TF target search^8,9^. Early dynamic studies of TFs relied heavily on Fluorescence Recovery After Photobleaching (FRAP)^10^. Recently, advanced fluorescence microscopy and labelling with organic dyes has permitted single molecule tracking (SMT) of TFs in live cells^8^. These techniques provide key dynamic information such as diffusion coefficients, residence times and evidence for different sub-populations (e.g. specific vs non-specifically bound). Fluorescence Fluctuation Spectroscopy (FFS) based methods also provide information on TF dynamics^9^. For example, Raster Image Correlation Spectroscopy (RICS) uses the raster scan of a confocal laser scanning microscope to gather information on the spatiotemporal dynamics of a fluorescent protein^11–13^. Similar to FRAP and SMT, RICS is a powerful technique to measure TF mobility in living cells^14^, for example detection by RICS of a reduced diffusion coefficient for transcriptionally active molecules can be verified via the use of DNA binding mutants^12^.

NF-Y is a highly-conserved TF that binds to the CCAAT consensus motif (the CCAAT-box) that is found at approximately one third of mammalian promoters, 60–100 bp upstream of the transcriptional start site (TSS)^15,16^. The genes driven by these CCAAT promoters are usually associated with ‘housekeeping’ roles and NF-Y binds to them in a largely cell-type invariant manner^17^. Analysis of DNase profiles in mouse embryonic stem cells (mESCs) identified NF-Y as a directional pioneer^7,18^. ChIP-seq results showed that the presence of NF-Y is anticorrelated with nucleosome occupancy^17^, which suggests that NF-Y can compete with nucleosome binding at CCAAT-box-containing loci. Indeed, NF-Y was recently shown to maintain the nucleosome free region (NFR) at CCAAT promoters and in doing so, define correct positioning of the TSS for these genes^19^. Due to this organising role at CCAAT promoters^20^, NF-Y can be considered a general transcription factor (GTF), similar to TATA binding protein (TBP) at TATA promoters.

Remarkably, in addition to its basal role at CCAAT promoters, NF-Y is also found at CCAAT-boxes at enhancers, repetitive elements and silent chromatin regions^17,20^. While binding of NF-Y at CCAAT promoters is cell-type invariant, NF-Y binding at distal enhancers is cell-type specific and occurs concurrently with binding of pioneer TFs, for example Oct4 and Sox2 in ESCs^17^. Furthermore, NF-Y displays early binding to promoters and enhancers during mouse preimplantation development (2–8 cell stage), where it contributes to zygotic genome activation^21^. These data suggest a second role of NF-Y in collaborating with cell-type specific pioneer TFs to establish and reinforce an open chromatin state at *cis* regulatory sequences of genes important for cell identity.

NF-Y is a heterotrimer composed of three subunits, NF-YA, NF-YB and NF-YC (herein called YA, YB and YC). YB and YC dimerise through conserved histone fold domains (HFD), which are structural homologs of the HFDs of histones H2B/H2A. In the NF-Y crystal structure, the YB/YC dimer binds DNA non sequence-specifically via a similar mode to H2B/H2A. Sequence-specific DNA binding is provided by the YA subunit, which trimerises with YB/YC and directs high affinity binding to the CCAAT-box in the DNA minor groove, resulting in an 80° DNA bend^22,23^. Despite this abundance of structural, biochemical and genomic data, to our knowledge there are only two published studies of fluorescently tagged NF-Y subunits in mammalian cells: (1) a microscopy study on the intrinsic nuclear localization of YA and YB, as well as the YB-mediated nuclear import of YC^24^ and (2) a FRET study suggesting the association of YB-CFP to YC-YFP^25^. Thus, the presence of the NF-Y trimer within the nucleus of a living cell has not yet been shown, nor are there any reports on the mobility of its subunits in the nuclear environment. To address these points, here we employed Fluorescence Lifetime Imaging Microscopy of Förster Resonance Energy Transfer (FLIM-FRET), Number and Brightness (NB), FRAP and RICS to study NF-Y dynamics in live cell nuclear architecture. Our FLIM-FRET and NB results confirmed the presence of the NF-Y trimer in live cells, while FRAP, RICS and biochemical experiments suggested the trimeric form to be the active, DNA bound species.

## Results

### NF-Y forms a heterotrimer in live cell nuclei

Throughout the paper, we transfected HeLa cells for 24 h with plasmids expressing fluorescently tagged NF-Y subunits in the presence versus absence of untagged NF-Y subunits and then performed fluorescence microscopy measurements on these samples to probe NF-Y trimer formation and dynamics. Within the transfected population of cells, where the transfected NF-Y subunits are present in excess to their endogenous counterparts, we selected cells exhibiting medium to low eGFP and mCherry fluorescence (Fig. S1), as this condition most accurately reflects NF-Y biology and is a requirement of FFS based methods of analysis (e.g. NB and RICS)^9,26^. Consistent with previous results, under this condition, nuclear localisation of YB and YC required co-transfection of both YB and YC whereas YA showed proper localisation when transfected alone (Fig. S1)^24^. Therefore, unless stated otherwise, all three NF-Y subunits were transfected (either tagged with eGFP, mCherry or not) to maintain proper NF-Y subunit localisation.

**Figure 1:**
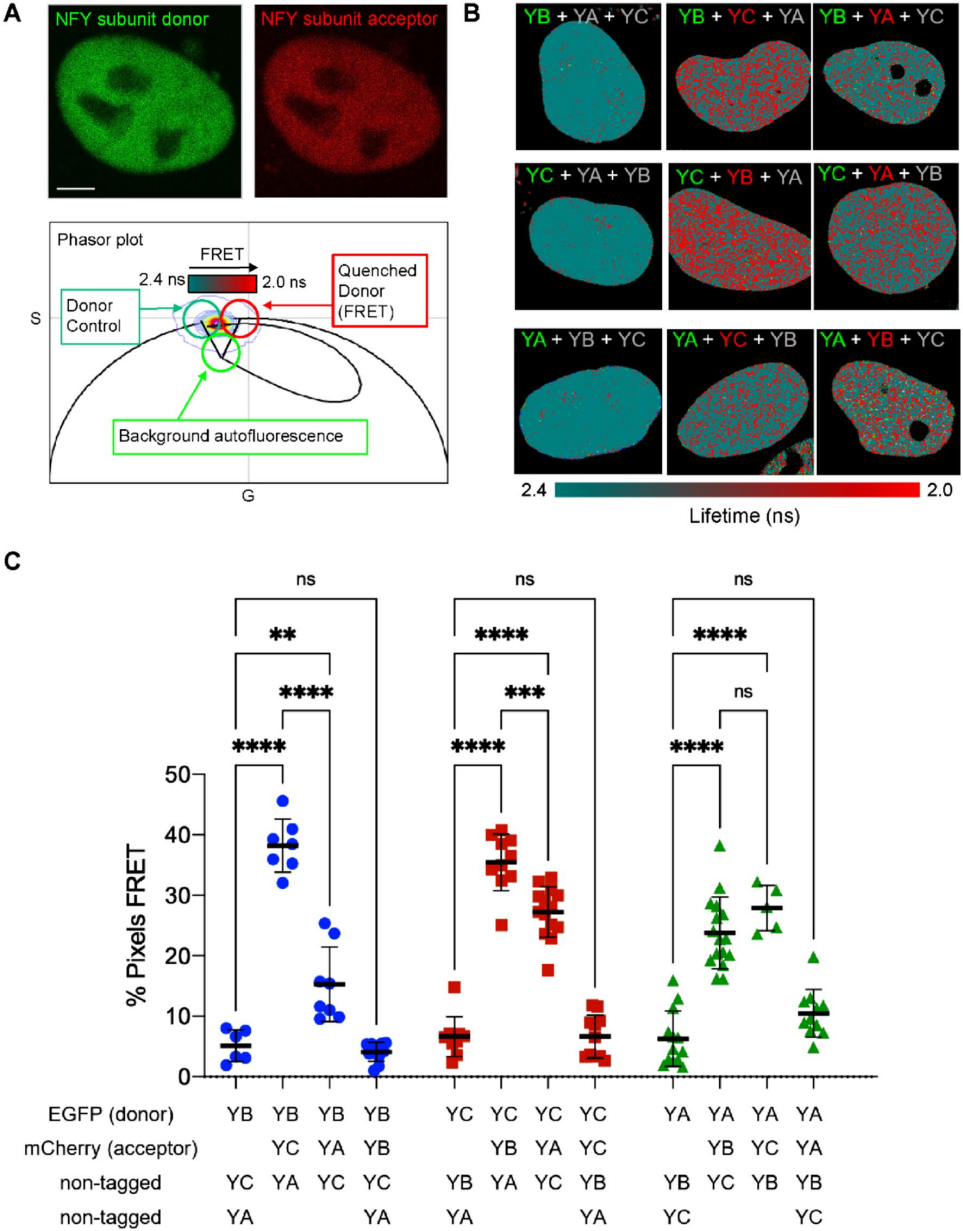
FLIM-FRET confirms the presence of nuclear heterotrimeric NF-Y. (**A**) Intensity image of an NF-Y sub-unit tagged to eGFP (donor molecule) versus mCherry (acceptor molecule) and phasor based FLIM analysis of FRET between two NF-Y subunits. Scale bar = 5 μm. When FRET occurs the lifetime of eGFP is decreased and in the phasor plot this results in a right shift along the universal circle. Cursors are positioned to highlight the pixels within the FLIM image that contain un-quenched donor (green circle) or FRET (red circle). Superimposed over the green and red cursors is the FRET trajectory which identifies the FRET state as having an efficiency of 16% (Fig. S2A). (**B**) Representative FLIM-FRET images of HeLa cells transfected with the indicated NF-Y subunits pseudo-coloured according to the cursor positions shown in (A). NF-Y subunits are indicated by green, red and grey text respectively for eGFP, mCherry and non-tagged. (**C**) Quantification of FLIM-FRET images from multiple cells transfected with the indicated NF-Y subunits. Mean and SD shown with unpaired t-tests.

To first show that transfected fluorescently-tagged NF-Y subunits interact and form complexes in live cell nuclei, we employed the phasor approach to FLIM analysis^27–29^ and measured FRET (FLIM-FRET) between eGFP and mCherry tagged NF-Y subunits (Fig. 1A). In each case, the efficiency of the FRET interaction between the different NF-Y subunits (e.g. eGFP-YB to mCherry-YC and eGFP-YB to mCherry-YA) was found to be approximately 16% (Fig. S2A) and it was the fraction of FRET detected between YB and YC versus YB and YA that varied (Fig. 1B,C). Specifically, a significantly higher fraction of eGFP-YB molecules throughout the nucleus undergo FRET with mCherry-YC (38.2 ± 4.4% pixels) than with mCherry-YA (15.3 ± 6.2% pixels). Consistent with this result, a significantly higher fraction of eGFP-YC undergoes FRET with mCherry-YB than it does with mCherry-YA and the fraction of eGFP-YA that undergoes FRET with mCherry-YC and mCherry-YB was equivalent to the fraction of FRET detected between eGFP-YB or eGFP-YC with mCherry-YA (Fig. 1B,C). Interestingly, for cells transfected with only two NF-Y subunits, although eGFP-YB gave robust FRET to mCherry-YC, neither eGFP-YB nor eGFP-YC gave FRET to mCherry-YA (Fig. S2B,C). Collectively, this result suggests that while YB and YC can form a dimer, YA/YB and YA/YC dimers cannot form, and YA interaction with YB or YC relies on the presence of both these subunits for NF-Y complex formation.

**Figure 2:**
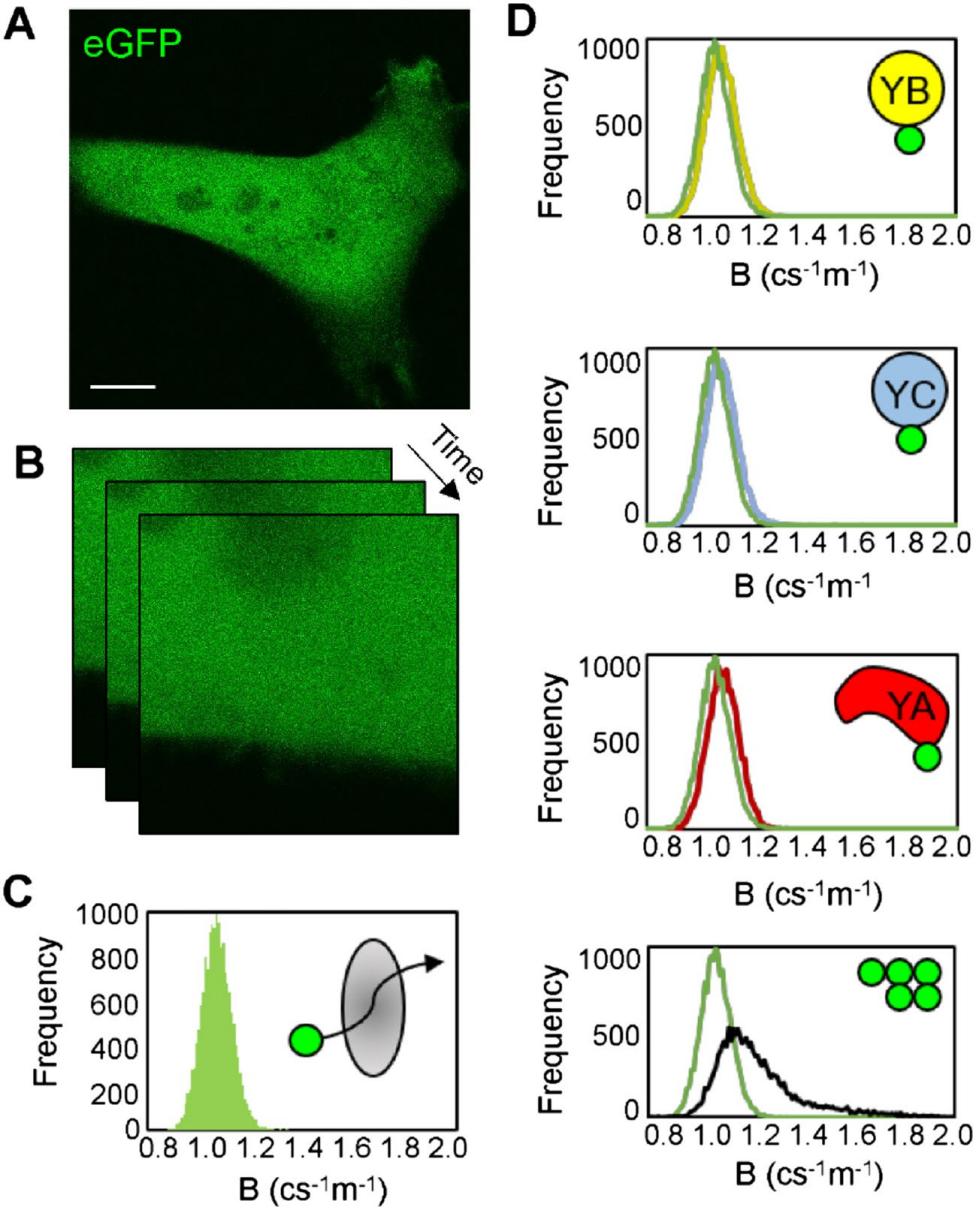
Number and Brightness (NB) analysis of NF-Y (**A**–**B**) A confocal image of a HeLa cell expressing eGFP (**A**) and the region of interest (ROI) selected for acquisition of an NB data set (**B**), which essentially is a time series of intensity frames optimised to capture fluctuations in eGFP fluorescence. Scale bar = 10 μm. (**C**) A moment analysis of the eGFP NB data set (**B**) results in a brightness histogram that reports the apparent brightness of eGFP—our monomeric control for determining the oligomeric stage of eGFP tagged NF-Y constructs. (**D**) An overlay of eGFP’s brightness histogram (green) with the brightness histogram of eGFP-YB (yellow), eGFP-YC (blue) and eGFP-YA (red), versus 5GFP (black), that is a positive control for oligomerisation (from top to bottom).

To determine the stoichiometry of the NF-Y complex that was indirectly measured by consecutive FLIM-FRET detection of YA/YB/YC interaction in live cells, we next transfected HeLa cells with eGFP-YA, eGFP-YB or eGFP-YC and measured their respective oligomeric states via Number and Brightness (NB) analysis (Fig. 2A–C)^30,31^. From comparison of the apparent brightness of eGFP-YB, eGFP-YC and eGFP-YA with the apparent brightness of our monomeric control eGFP we find each NF-Y subunit to be monomeric (Fig. 2D). This result alongside the NF-Y FLIM-FRET experiments (Fig. 1), suggest that the dominant NF-Y complex in live cells has single copies of each subunit, i.e. a trimeric complex with 1:1:1 stoichiometry. Also, in support of this NB proposed stoichiometry for the NF-Y complex, no FRET was observed between a single NF-Y subunit (i.e. EGFP-YA and mCherry-YA, EGFP-YB and mCherry-YB or EGFP-YC and mCherry-YC) (Fig. 1C, columns 4, 8 and 12), which argues against NF-Y complexes containing homodimers of its subunits. Taken together, our FRET and NB data strongly suggests that NF-Y in the cell nucleus forms a heterotrimer composed of YA, YB and YC, and not a higher order complex containing multiples of NF-Y subunits.

### A trimer of NF-Y likely binds chromatin via YA

**Figure 3:**
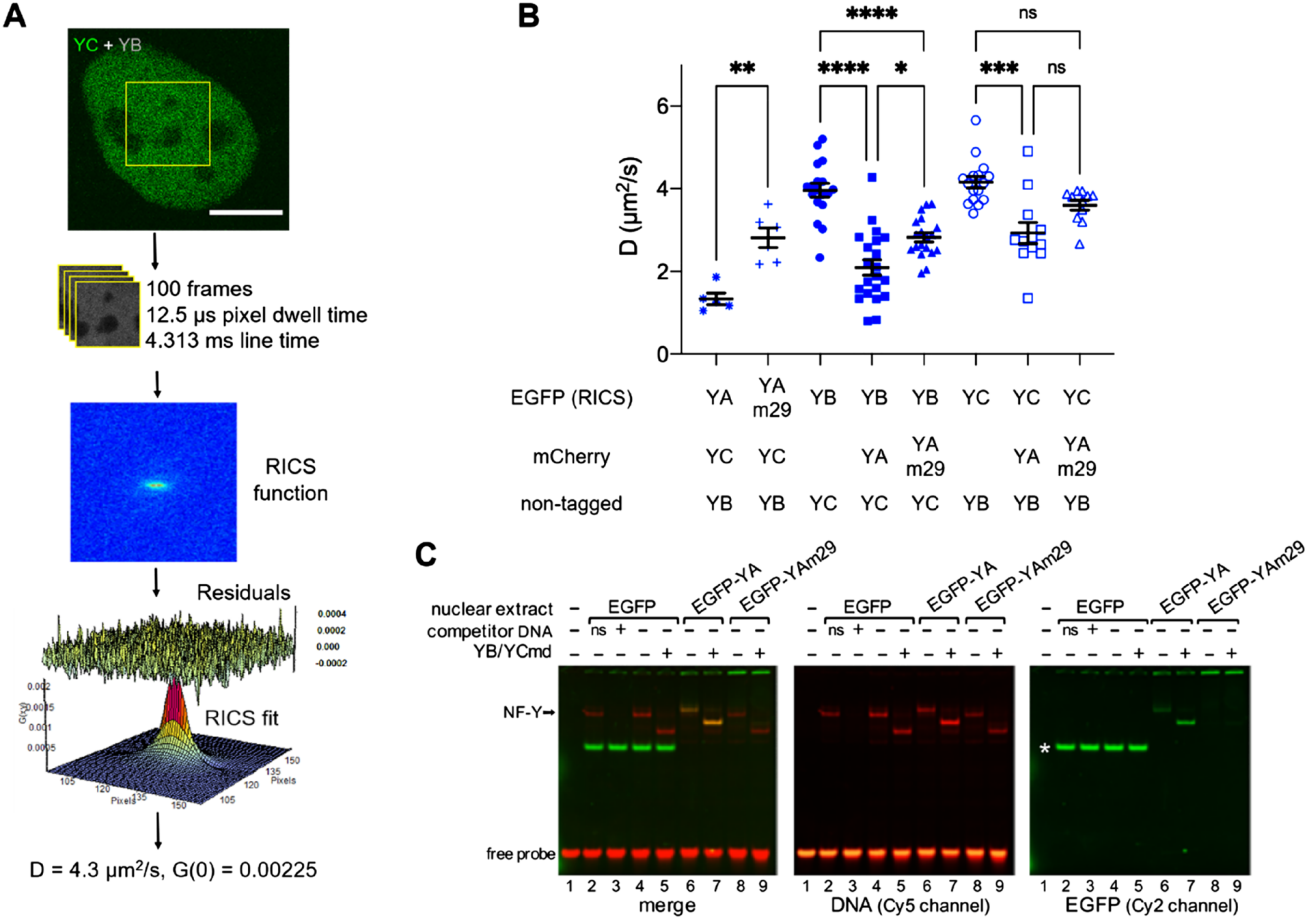
RICS analysis shows YB/YC require trimerisation with YA for NF-Y chromatin binding. (**A**) A confocal image of a HeLa cell expressing eGFP-YC in the presence of dark YB and the region of interest (ROI) selected for acquisition of a RICS data set (top panel). Scale bar = 10 μm. Spatiotemporal correlation of the recorded fluctuations in eGFP-YC fluorescence intensity results in a RICS profile that is fit to a 1-component 3D diffusion model in SimFCS (bottom panel). (**B**) The apparent diffusion coefficient (ensemble mobility) extracted by RICS of the different EGFP-tagged NF-Y proteins in HeLa cells. Data are Mean ± SD combined from two separate days. Note that the mCherry tag on YA was not used for RICS measurements, but only to confirm the presence of the transfected protein in the cells analysed. *p < 0.05, **p < 0.01, ***p < 0.001, ****p < 0.0001 according to one-way ANOVA followed by Tuckey’s multiple comparison test. (**C**) Fluorescent EMSA with nuclear extracts of HeLa cells transfected with EGFP-YA constructs. Binding of the endogenous NF-Y to the CCAAT-probe was challenged either with a CCAAT-competitor or a non-specific (ns) oligo (lanes 2–3). The arrow indicates the migration of endogenous NF-Y-bound probe. When indicated, recombinant minimal domain YB/YC dimer (YB/YCmd) was included in reaction (lanes 5, 7, 9), reconstituting a down-shifted complex with endogenous or overexpressed YA. The white asterisk (*) indicates free EGFP protein migration in the gel in empty-vector control samples. Note that the slightly retarded gel mobility of the complex containing EGFP-YA is due to the EGFP-tag.

To investigate the diffusive behaviour of NF-YA, YB and YC in live cells we next employed RICS (Fig. 3A), which measures the diffusion coefficient and concentration of fluorescently-tagged proteins expressed in live cells^11,13^. The expectation was that the DNA-bound state of the NF-Y complex would give rise to a slow-diffusing sub-population that is temporally discrete and NF-Y mobility would be described by a 2-component diffusion or diffusion-binding model. However, given that our NF-YA, YB, and YC RICS data fit a 1-component diffusion model, and the extracted diffusion coefficient was sensitive toward the ensemble mobility of slower diffusing sub-populations specific to each NF-Y subunit measured, we interpreted this finding to suggest that the NF-Y complex, like a subset of transcription factors, undergoes a continuum of DNA binding affinities across multiple timescales (mobile to immobile)^32^. This interpretation was supported by the fact that the ensemble mobility of the DNA binding subunit eGFP-YA (1.33 ± 0.31 μm^2^/s) was slower than the eGFP-YAm29 DNA binding mutant (2.81 ± 0.58 μm^2^/s), which harbors a triple amino acid substitution within the base-readout subdomain and renders it unable to bind CCAAT-boxes throughout the genome^33^.

With this rationale behind fitting our RICS data in mind, we found the ensemble mobility of YB versus YC in the absence of YA (i.e. cells transfected with eGFP-YB and dark YC or eGFP-YC and dark YB, but not YA) to be approximately ~ 4 μm^2^/s (eGFP-YB = 3.96 ± 0.69 μm^2^/s and eGFP-YC 4.16 ± 0.55 μm^2^/s) (Fig. 3B), and remarkably, when YB versus YC are in the presence of YA (i.e. mCherry-YA co-transfected), we detect a significant reduction in the ensemble mobility of these two species (eGFP-YB = 2.09 ± 0.86 and eGFP-YC 2.93 ± 0.89 μm^2^/s respectively) (Fig. 3B). Importantly, across this panel of transfected cells measured by RICS, we found the ensemble mobility extracted for each NF-Y subunit was not correlated with protein concentration (R = − 0.19) (Fig. S3A) and the trends were recapitulated by a series of analogous FRAP experiments which also demonstrate YA to slow down YB and YC (Fig. S4). This result, alongside our FLIM-FRET detection of a significant fraction of YB being in complex with YC, suggests that the YB/YC dimer requires complex formation with YA in order to bind DNA. In support of this finding, co-transfection of YB and YC with instead the DNA binding mutant YAm29, resulted in a less drastic reduction in the ensemble mobility of these two species (2.82 ± 0.48 and eGFP-YC = 3.60 ± 0.40 μm^2^/s respectively) (Fig. 3B).

To verify that the eGFP-tagged version of YA can become part of a DNA-binding competent NF-Y complex, as suggested by the RICS and FRAP data, we performed an EMSA with nuclear extracts from transfected HeLa cells (Fig. 3C). Indeed, this experiment found that the NF-Y specific endogenous complex was completely substituted by one containing eGFP-YA (Fig. 3C, lane 4 vs lane 6). Moreover, the addition of a recombinant YB/YC version containing only the histone-fold domain (which allows discrimination from endogenous YB/YC by size on the gel) increased the DNA-bound fraction in EGFP-YA transfected cells extract (lane 7), suggesting that the transfected eGFP-YA is in excess with respect to endogenous YB/YC under these conditions. As expected, the eGFP-YAm29 mutant, albeit comparable in expression to the wild-type version (Fig. S3B), showed negligible DNA-binding activity (lanes 8–9). Finally, we also confirmed that co-transfected YA slows down EGFP-YB in immortalised non-transformed human retinal RPE-1 cells (Fig. S3C), thereby generalising this result beyond cells with heavily-altered genomes such as HeLa.

## Discussion

Here we have applied FLIM-FRET, NB, RICS and FRAP to investigate the live cell dynamics of the NF-Y transcription factor. Overall, our results suggest that NF-Y moves and acts as a trimeric complex in the nucleus. The consecutive detection of FRET between all possible pairs of the three NF-Y subunits strongly suggests YA/YB/YC complex formation, while the monomeric brightness of each subunit measured by NB supports the presence of a trimeric NF-Y complex in live cell nuclei. Also, the lack of FRET between the same NF-Y subunits argues against higher order complexes containing multiples of each subunit. Our RICS data, in agreement with FRAP, suggest that the trimeric NF-Y complex is competent for DNA binding and this interaction is mediated by the CCAAT-recognition moiety of the YA DNA binding domain. Specifically, the fitting of our RICS data to a 1-component 3D diffusion model and the extracted diffusion coefficients—interpreted as a readout of NF-Y subunit ensemble mobility in the presence of a continuum of DNA binding affinities—showed that YB/YC are slower when YA, the DNA-recognition subunit, is additionally co-expressed. This result suggests that our exogenously expressed NF-Y has the ability to bind widely to chromatin. Experiments comparing NF-Y mobility via RICS and single molecule tracking^8^ would provide further insight into the nature of the NF-Y diffusive sub-populations that result from DNA binding. The structural homology between the HFD of YB/YC and H2A/H2B has led to the suggestion that YB/YC may displace H2A/H2B at certain nucleosomes to form ‘hybrid nucleosomes’^23^. Whether binding of exogenously expressed NF-Y occurs at canonical NF-Y binding locations, other CCAAT-box-containing loci, or as part of a YA-containing hybrid nucleosome is unknown but could be investigated using ChIP-seq to the eGFP-tag.

So how does the cell modulate NF-Y activity? Since exogenously expressed YA alone still shows a slow diffusion coefficient (Fig. S3C), it is likely that YB/YC are naturally in excess. A limiting amount of YA would allow the cell to control NF-Y activity by adjusting the level of YA only. However, we observed that overexpression of YA alone does not increase the DNA-bound fraction in EMSAs performed with nuclear extracts (Fig. 3C), suggesting that the endogenous trimer is well-balanced. Rather, EGFP-YA may substitute its endogenous version, maintaining a similar DNA-bound fraction, which only increases after exogenous addition of more YB/YC dimer (Fig. 3C, lane 6 vs lane 7). Although the EMSA approach validates the DNA-binding properties of fluorescently tagged YA, it still represents a bulk measurement where protein stoichiometries from the whole cell population are averaged. This issue is particularly relevant with findings that several types of cancers—mostly epithelial—have increased levels of YA mRNAs, compared to normal tissues ^34–38^. This is unlike YB/YC mRNA species, which remain constant in most cancers. A characterization of the actual levels of the subunits in cancer specimens is required to understand this point. Similarly, YA decreases under certain conditions, such as in myoblast-to-myotube differentiation^39^. Further RICS experiments in different cell types (e.g. along a differentiation time-course) could investigate the intriguing role of NF-Y at lineage-specific enhancers^17^. In summary, the FRET, NB and RICS data presented here provides unique insights into NF-Y dynamics in live cells and provides a prototypical example for combinatorial studies of other multimeric proteins.

## Materials and methods

### Cloning of NF-Y constructs

N-terminal tagged constructs EGFP-YA (37 kDa isoform), EGFP-YB and EGFP-YC (37 kDa isoform) were described in^24^. To generate EGFP-tagged YAm29 mutant, the mouse NF-YA coding sequence harboring the triple substitution R311A, G312A, E313A from pSG5-YAm29 construct (*EcoRI-BglII*) was cloned into pEGFP-C1 vector (*EcoRI-BamHI*). mCherry-YA (N-terminal tag, 34 kDa isoform) was obtained by subcloning NF-YA coding sequence from pSG5-YA (*EcoRI-BglII*)^40,41^ into pmCherry2-C1 (*EcoRI-BamHI*), through restriction ends ligation. mCherry-YB was generated by subcloning NF-YB coding sequence from pCMV2-flag-YB^22^ into pmCherry2-C1 using *KpnI-SmaI* restriction sites. mCherry-YC (37 kDa isoform) was obtained from pSG5-YC construct^41^ by subcloning NF-YC coding sequence into pmCherry2-C1 vector using *KpnI-BamHI* sites. mCherry2-C1 was a gift from Michael Davidson (Department of Biological Science, Florida State University, Tallahassee, FL, USA) (Addgene plasmid # 54563). All constructs were verified by sequencing.

### Cell culture and transfection

HeLa cells were grown (T25 flasks) and imaged in DMEM (lonza) with 10% FBS (Life Technologies) and pen/strep (Life Technologies). RPE-1 cells were grown in DMEM:F12 with 10% FBS, pen/strep and 10 μg/mL hygromycin. On day 1, cells were seeded onto 8-well chamber slides (ibidi μ-Slide, 8-well with ibiTreat) (15,000 cells) or 35 mm glass bottom dishes (FluoroDish FD35, World Precision Instruments, Inc) (150,000 cells). On day 2, cells were transfected with plasmids expressing untagged and eGFP or mCherry tagged NF-Y subunits using Lipofectamine 3000 (Thermo Fisher). Transfection mix consisted of 200 ng of each plasmid (with 2 μL P3000 reagent per 1 μg of DNA) made up to 50 μL with PBS then mixed with 1.5 μL Lipofectamine 3000 reagent per 1 μg DNA made up to 50 μL in PBS for 5 min. 100 μL of this mix was added to cells in 2 mL of media in 35 mm dishes or 15 μL to cells in 300 μL media in each well of 8-well chamber slides. On day 3, cells were imaged by FLIM-FRET, NB, RICS or FRAP.

### Fluorescence lifetime imaging microscopy (FLIM) of Förster resonance energy transfer (FRET)

FLIM-FRET data was acquired on an Olympus FV3000 confocal laser scanning microscope coupled to a 488 nm pulsed laser operated at both 20 and 80 MHz (ISS) and an ISS A320 Fast FLIM box. This excitation source enabled selective excitation of the donor fluorophore (eGFP) in each FRET experiment and a 405/488/561 dichroic mirror was used to separate this fluorescent signal from the laser light. The donor fluorescence was then reflected by a 550 nm long pass filter, collected by a photomultiplier detector (H7422P-40 of Hamamatsu) fitted with a 520/25 nm bandwidth filter and processed by the FastFLIM box data acquisition card. All FLIM-FRET data were acquired by the ISS Vista Vision software that pre-calibrates the instrument and phasor space against a known reference lifetime (we use fluorescein at pH 9 which has a single exponential lifetime of 4.04 ns) and processed by the SimFCS software developed at the Laboratory for Fluorescence Dynamics (LFD). A 60X water immersion objective 1.2 NA was used, and the cells were imaged at 37 degrees in 5% CO_2_.

FRET was quantified within each donor FLIM image by the phasor approach to lifetime analysis, where, as described in previously published papers, the donor fluorescence lifetime recorded in each pixel of a FLIM image is described by a *g* and *s* coordinate (phasor) presented in a phasor plot^27–29^. In pixels where donor molecules undergo FRET with acceptor molecules, the phasor coordinate is right shifted along a curved trajectory that is described by the classical definition of FRET efficiency^42,43^. To determine the efficiency of the FRET state, the phasor coordinates of the unquenched donor and background autofluorescence were first determined independently and then a FRET trajectory was extrapolated from this baseline. In the case of FLIM-FRET experiments based on eGFP-YB, for example, the unquenched donor lifetime of this construct was found to be approximately ~ 2.4 ns (0% FRET), and from superimposition a of FRET trajectory defined by this calibration over the phasor distribution of eGFP-YB in the presence of mCherry-YC or mCherry-YA, the most quenched lifetime was detected at ~ 2.0 ns, which corresponds to a 16% FRET efficiency (Fig. S2A). By placing a cursor at these two phasor locations (cursor 1_donor_ = 2.4 ns and cursor 2_FRET_ = 2.0 ns), the fraction of FRET and spatial distribution of each NF-Y subunit interaction was quantified at a single cell level. All FLIM-FRET data analysis was performed in the SimFCS software developed at the Laboratory for Fluorescence Dynamics (LFD). Statistical analysis was performed in Graphpad Prism 8.0.

### Number and brightness (NB)

NB data was acquired on the Olympus FV3000 confocal laser scanning microscope coupled to an ISS A320 Fast FLIM box via use of a solid-state laser diode operating at 488 nm. This excitation source enabled excitation of each eGFP tagged NF-Y sub-unit, as well as the monomeric (eGFP) and oligomeric (5GFP) controls required for calibration of NB analysis. The resulting fluorescence signal was directed through a 405/488/561 dichroic mirror to a photomultiplier detector (H7422P-40 of Hamamatsu) fitted with an eGFP 500/25 nm bandwidth filter. All NB data acquisitions employed a 60X water immersion objective 1.2 NA and involved selecting a 10.6 μm region of interest (ROI) within a HeLa cell nucleus transfected with eGFP, 5GFP or eGFP-YA/YB/YC. A time series of intensity frames (n = 100) was acquired within this ROI at 37 degrees in 5% CO_2_, which for a 256 × 256 pixel frame size and 12.5 µs pixel dwell time, resulted in a line time of 4.313 ms and a frame time of 1.108 s.

The apparent brightness of each eGFP tagged NF-Y subunit was extracted from each NB data set via a moment-based analysis and translated into oligomeric state to determine the stoichiometry of YA/YB/YC in the NF-Y complex. As described in previously published papers^32,33^, for a given intensity fluctuation that has an average intensity (first moment) and a variance (second moment), the ratio of these two properties describes the apparent brightness (B) of the molecules that give rise to the intensity fluctuation. The true molecular brightness (*ɛ*) of the molecules is related to the measured apparent brightness (*B*) by $\mathrm{B}=\upvarepsilon +1$, where 1 is the brightness contribution of our photon counting detector. Calibration of the apparent brightness of monomeric eGFP enabled extrapolation of the expected apparent brightness of an eGFP dimerisation or oligomerisation. An overlay of the apparent brightness histogram for monomeric eGFP with the apparent brightness histogram for eGFP-YA, eGFP-YB and eGFP-YC, however, revealed no significant difference. Thus, the apparent brightness histogram of 5GFP was used as a positive control. Artefact due to cell movement or photobleaching were subtracted via use of a moving average algorithm. All brightness calculations were carried out in SimFCS from the Laboratory for Fluorescence Dynamics (LFD).

### Raster image correlation spectroscopy (RICS)

RICS data was acquired on the Olympus FV3000 confocal laser scanning microscope via use of a solid-state laser diode operating at 488 nm. This excitation source enabled excitation of each eGFP tagged NF-Y sub-unit and the resulting fluorescence signal was directed through a 405/488/561 dichroic mirror to an internal GaAsP photomultiplier detector set to collect 500–550 nm. All RICS data acquisitions employed a 60 × water immersion objective 1.2 NA and involved selecting a 10.6 μm region of interest (ROI) within a HeLa cell nucleus transfected with eGFP-YA/YB/YC at a low to medium expression level. A time series of intensity frames (n = 100) was acquired within this ROI at 37 degrees in 5% CO_2_, which for a 256 × 256 pixel frame size resulted in a pixel size of 41 nm and for a pixel dwell time set to 12.5 µs this scan rate resulted in a line time of 4.313 ms and a frame time of 1.108 s.

The apparent diffusion coefficient of each eGFP tagged NF-Y subunit was extracted from each RICS data set (n = 100 frames) via application of the RICS function that, as described in previously published papers^11,44^, calculates the average autocorrelation of each frame after a moving average background subtraction (n = 10 frames). The resultant 3D RICS profiles describing eGFP-YA versus eGFP-YB or eGFP-YC mobility were then fit to a 1-component diffusion model, and the recorded diffusion coefficient, interpreted to report the average rate of NF-Y subunit diffusion that results from a continuum of DNA binding affinities with chromatin^32^. All RICS data analysis was carried out in SimFCS from the Laboratory for Fluorescence Dynamics (LFD). Statistical analysis was performed in Graphpad Prism 8.0.

### Fluorescence recovery after photobleaching (FRAP)

FRAP data was acquired on the Olympus FV3000 microscope via use of a solid-state laser diode operating at 488 nm, the ‘stimulate’ function and an internal GaAsP photomultiplier detector set to collect 500–550 nm. This 488 nm laser diode enabled both excitation and bleaching of each eGFP tagged NF-Y sub-unit and the ‘stimulate’ function was set to: (1) record 5 pre-bleach frames at zoom 15 (~ 20 µm square) with a pixel frame size 128 × 128 and frame time of 350 ms, (2) bleach a ~ 30 × 30 pixel nuclear region of interest (ROI) within the pre-bleach frame (488 nm, 20% laser power, 100 μs pixel dwell), and (3) record a stream acquisition of 100 post-bleach frames at zoom 15 with the original pixel frame size of 128 × 128 and frame time of 350 ms. Bleach ROIs were assigned and measured in ImageJ (multi-measure) and exported data was fit to a single exponential recovery in Matlab, from which the half-time of fluorescence recovery was extracted. The fluorescence recovery half-time measured across the panel of NF-Y transfections (Fig. S4A,B) was compared to the diffusion coefficient extracted via RICS and as expected found to anti-correlate (R = − 0.81) (Fig. S4C,D).

### Electrophoretic mobility shift assay (EMSA)

EMSA was performed as described in^45^ and summarized here with few modifications. HeLa cells expressing EGFP-fusion constructs were harvested 24 h post-transfection to prepare nuclear extracts (NE). DNA-binding reactions were prepared in a binding mix containing 20 nM Cy5-labeled (5′-end) DNA probe designed from human HSP70 promoter CCAAT-box (CTTCTGAGCCAATCACCGAGCTCGATGAGGC). Where indicated, 60 nM recombinant NF-YB/YC minimal-domain dimer (YB/YCmd; YB aa 51–143, YC aa 27–120) was included in the reaction mix. For competition controls, a 50-fold excess of unlabeled DNA competitor, either containing the HSP70 CCAAT-box (TTCTGAGCCAATCACCGAGCTCGAT) or a non-relevant site (TTAGGCGCCCACGTGATCCTCCGA), was included in the reaction mix. For each reaction, 10 µg of the correspondent nuclear extract were used. After 30 min of incubation at 30 °C, an aliquot of each reaction was loaded on a 4.5% non-denaturing polyacrylamide gel, run in 0.25 × TBE at 100 V. Fluorescent image of the gel was acquired in multichannel modality (Cy5 and Cy2 channels) using a Chemidoc MP apparatus (Bio-Rad).

### Immunoblot

Nuclear extracts of transfected HeLa cells were loaded on a 10% SDS-PAGE gel added with 0.5% 2,2,2-trichloroethanol (TCE, Sigma-Aldrich) for stain-free protein detection^46^. After running, the gel was activated for one minute with UV light using a Chemidoc MP apparatus. Proteins were transferred to a nitrocellulose membrane and lanes loading visualized under UV light. Specific proteins were probed with the following antibodies: rabbit anti-NF-YA (H-209, Santa Cruz Biotechnology) and rabbit anti-NF-YB (GeneSpin), and HRP-conjugated secondary antibodies.

## Supplementary Information

Supplementary Information.
