# Supplementary material for: Live cell dynamics of the NF-Y transcription factor
Source: Sci Rep. 2021 May 26;11:10992. doi: 10.1038/s41598-021-90081-1 (PMC8155045; doi:10.1038/s41598-021-90081-1)
Supplement: Supplementary file 1 — Supplementary Information. [file 41598_2021_90081_MOESM1_ESM.pdf]

Supplementary Information

Live cell dynamics of the NF-Y transcription factor

David G. Priest, Andrea Bernardini, Jieqiong Lou, Roberto Mantovani, Elizabeth Hinde.

Supplementary Figures 1-7

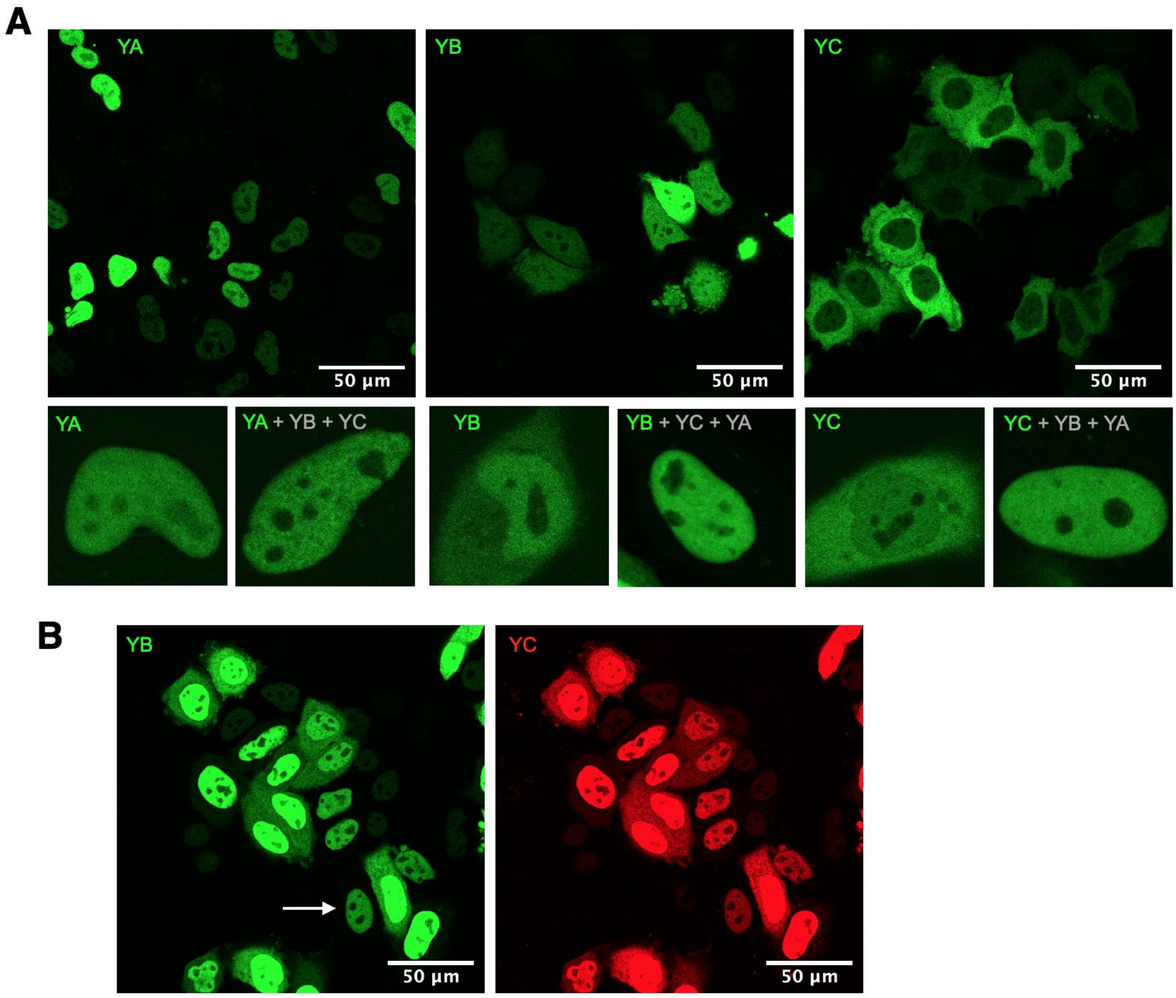

Figure S1. Confocal microscopy images of NF-Y subunit localisation in HeLa cells. (A) eGFP-YA transfected in HeLa cells alone shows a nuclear localisation (top left) and little changes upon co-transfection of dark YB and YC (small image below right). eGFP-YB transfected in HeLa cells alone shows a diffuse nucleo-cytoplasmic localisation (top middle) and co-transfection with dark YC results in eGFP-YB properly localising to the nucleus (small image below right). eGFP-YC transfected in HeLa cells alone is excluded from the nucleus (top right) and co-transfection with YB restores its correct nuclear localisation (small image below right). **(B)** HeLa cells expressing eGFP-YB (left) and mCherry-YC (right) highlight co-localisation and variable expression level. White arrow in eGFP-YB image indicates a cell with a medium-low nuclear-localised expression level that is suitable for FRET, NB or RICS imaging. Image contrast adjusted to highlight different expression levels.

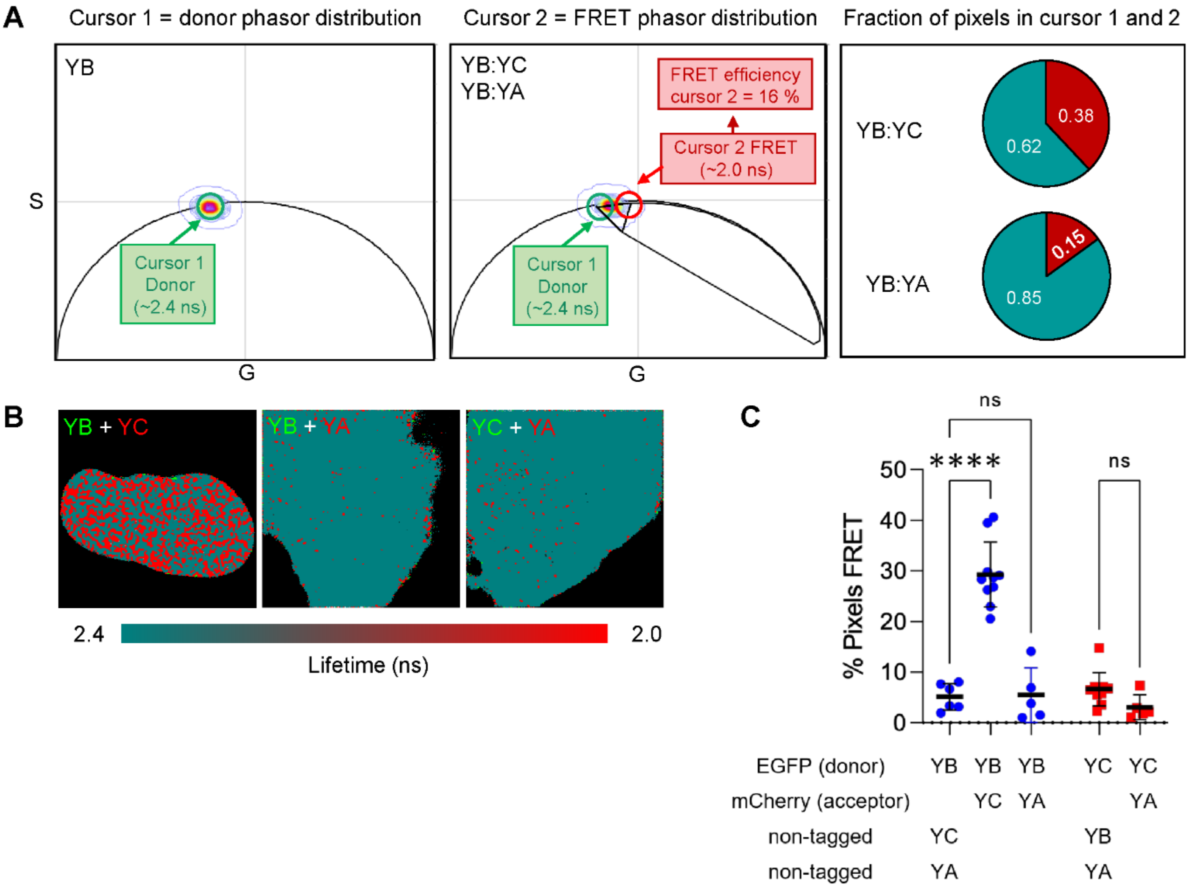

Figure S2. Additional FLIM-FRET analysis of NF-Y subunit complex formation. (A) The phasor coordinates of the unquenched donor lifetime (for example, eGFP-YB in the absence of an acceptor) (left panel) were first measured in live HeLa cells and used to define the cursor position (~ 2.4 ns) from which a FRET trajectory is extrapolated for FRET analysis of a quenched donor (for example, eGFP-YB in the presence of mCherry-YC or mCherry YA) (middle panel). We find from this FRET analysis that the phasor distribution of an eGFP tagged NF-Y subunit in the presence of a mCherry tagged NF-Y subunit extends to a 16 % FRET efficiency and so we place a FRET cursor centered at this phasor coordinate (~ 2.0 ns). The two phasor cursors (donor versus FRET) enable a fractional component analysis of each FLIM image and quantification of the percentage of pixels that give rise to a donor phasor (phasor coordinate within unquenched donor cursor) versus a FRET phasor (phasor coordinate with 16 % FRET cursor) (right panel). From this analysis we find that in the case of the YB to YC interaction 38.2 ± 4.4 % pixels exhibit FRET and in the case of the YB to YA interaction, 15.3 ± 6.2 % pixels. **(B)** Representative FLIM-FRET images of HeLa cells transfected with only two NF-Y subunits. The NF-Y subunits are indicated by the green and red text for eGFP and mCherry, respectively. The FLIM-FRET images are pseudo-coloured according to the donor (teal) and FRET phasor (red) cursors analogous to Figure 1B. **(C)** Quantification of FLIM-FRET images from multiple cells transfected with the indicated NF-Y subunits in (B). Mean and SD shown with unpaired t-tests. YB and YC donor-only controls are from Figure 1C.

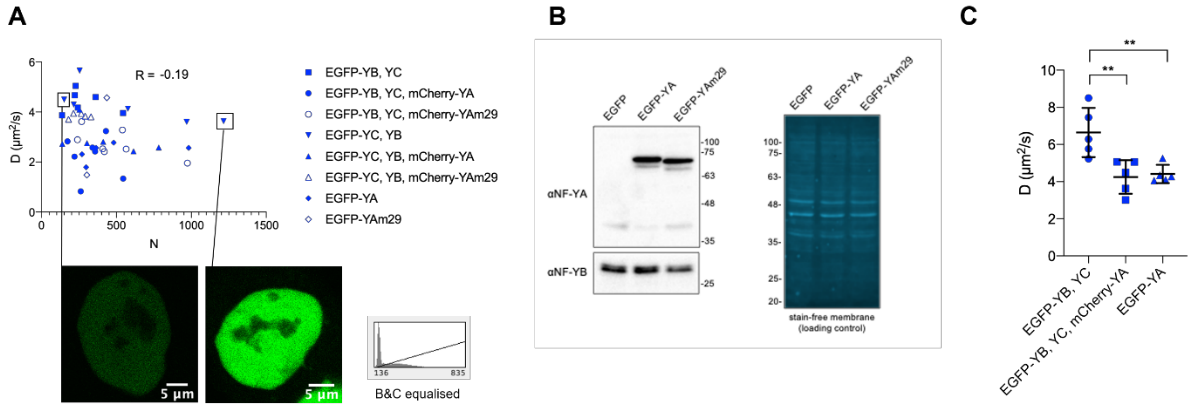

Figure S3. Additional RICS and EMSA data. (A) RICS diffusion coefficient is not correlated with expression level. Diffusion coefficients extracted from RICS data presented in Figure 3B plotted against the number of protein molecules (N). Two cells expressing eGFP-YC and YB are shown below with equalised brightness and contrast (136-835, 8-bit) to highlight the range of expression levels. **(B)** eGFP-YA construct expression levels in nuclear extracts used in EMSA. Immunoblot of nuclear extracts from transfected HeLa cells used in EMSA (left panel). Even protein loading was assessed on the membrane after electroblotting thanks to TCE-based stain-free protein detection protocol (right panel). **(C)** The slowing down of the RICS extracted diffusion coefficient measured for YB/YC in the presence of YA was confirmed in RPE-1 cells.

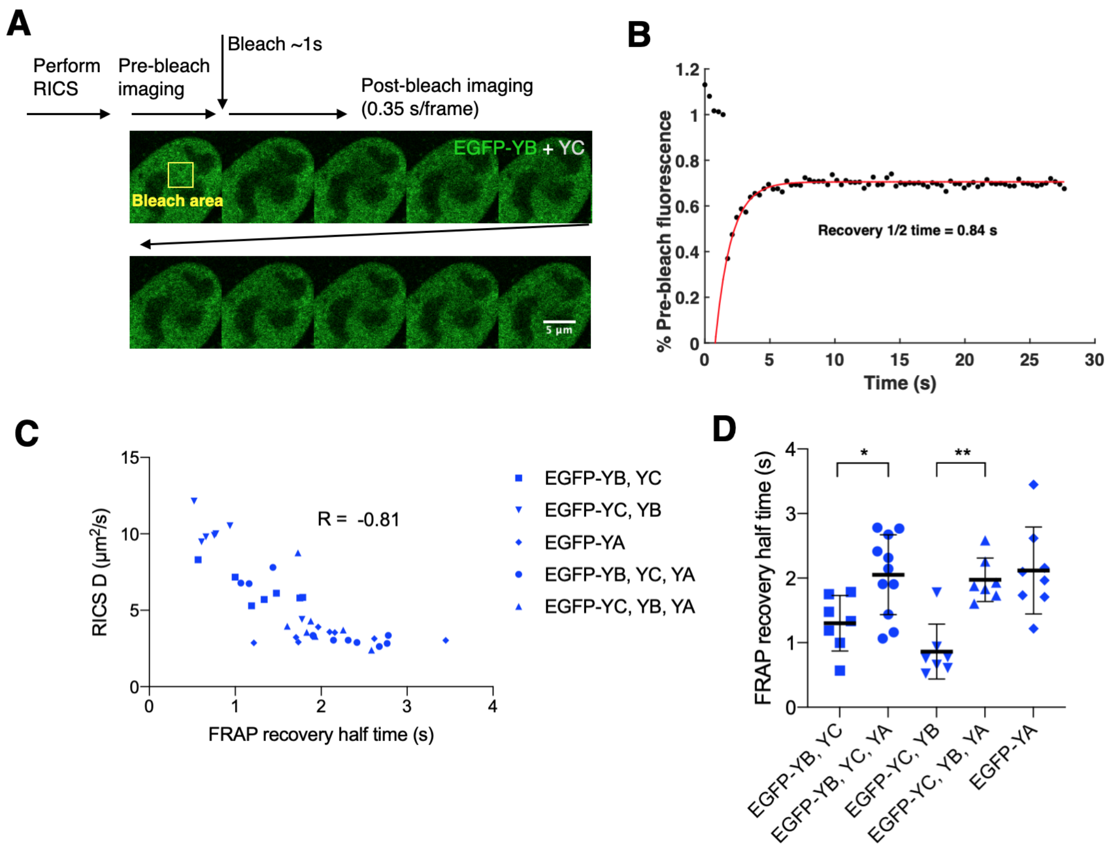

Figure S4. Verification of RICS obtained trends in NF-Y subunit mobility via FRAP. (A) Experimental sequence for the RICS and FRAP experiments that were performed on the same HeLa cells transfected with different NF-Y subunits. **(B)** The normalised fluorescence intensity profile of the FRAP experiment reported in (A) as a function of time (black) with a single component exponential fit superimposed (red). The fluorescence did not recover fully due to the loss of overall fluorescence from the photobleaching. **(C)** A plot of the RICS diffusion coefficient against the FRAP recovery half time measured in a single HeLa cell for the indicated NF-Y subunit combinations. **(D)** Fluorescence recovery half-times derived from FRAP experiments across the panel of NF-Y subunit transfections. * $p<0.05$, ** $p<0.01$ according to ordinary one-way ANOVA.

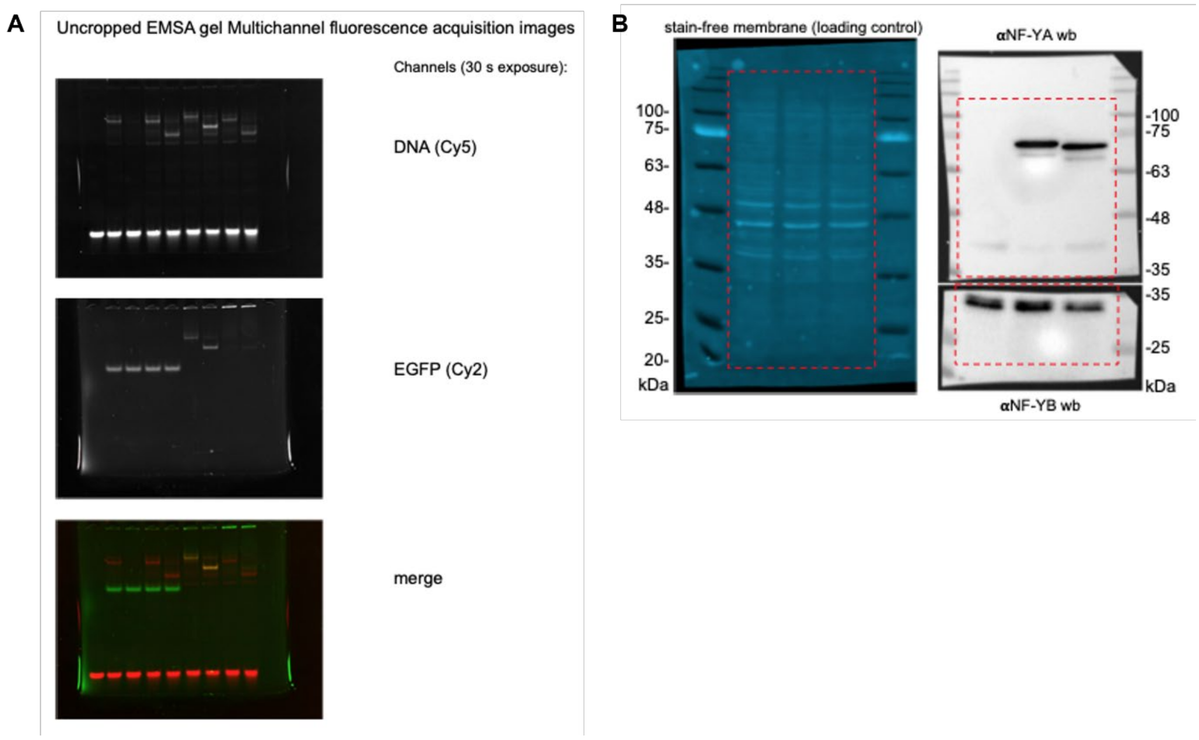

Figure S5. Uncropped biochemical data from Figure 3C. (A) Gel images. **(B)** Western blots.
